# Role of Tumor-Derived Chemokines in Osteolytic Bone Metastasis

**DOI:** 10.3389/fendo.2018.00313

**Published:** 2018-06-07

**Authors:** Salvatore J. Coniglio

**Affiliations:** New Jersey Center for Science, Technology and Mathematics, Kean University, Union, NJ, United States

**Keywords:** metastasis, bone, chemokines, chemokine receptors, CXCR4, breast carcinoma, prostate carcinoma, myeloma

## Abstract

Metastasis is the primary cause of mortality and morbidity in cancer patients. The bone marrow is a common destination for many malignant cancers, including breast carcinoma (BC), prostate carcinoma, multiple myeloma, lung carcinoma, uterine cancer, thyroid cancer, bladder cancer, and neuroblastoma. The molecular mechanism by which metastatic cancer are able to recognize, infiltrate, and colonize bone are still unclear. Chemokines are small soluble proteins which under normal physiological conditions mediate chemotactic trafficking of leukocytes to specific tissues in the body. In the context of metastasis, the best characterized role for the chemokine system is in the regulation of primary tumor growth, survival, invasion, and homing to specific secondary sites. However, there is ample evidence that metastatic tumors exploit chemokines to modulate the metastatic niche within bone which ultimately results in osteolytic bone disease. In this review, we examine the role of chemokines in metastatic tumor growth within bone. In particular, the chemokines CCL2, CCL3, IL-8/CXCL8, and CXCL12 are consistently involved in promoting osteoclastogenesis and tumor growth. We will also evaluate the suitability of chemokines as targets for chemotherapy with the use of neutralizing antibodies and chemokine receptor-specific antagonists.

## Introduction

Cancer is the second leading cause of death in the developed world. Metastatic spread of tumor cells to vital organs results in mortality and morbidity (1, 2). The metastatic process is complex and involves genetic alterations of the cancer cells as well as interaction with the tumor microenvironment (3–6). The multi-step process of the metastatic cascade includes local invasion of the tumor followed by entry into blood vessels (intravasation), survival of circulating tumor cells (CTCs) in the blood (CTCs), adhesion and exit of cancer cells from blood vessels (extravasation) and invasion, colonization and outgrowth of the primary cancer in distal secondary organs. Each stage requires close collaboration of cancerous cells with specific elements of the microenvironment.

Clinicians and researchers have noted for over a century that metastasis is not a random process and that specific cancers tend to predominantly metastasize to certain organs. The “seed vs soil” hypothesis first put forth by the physician Stephen Paget to explain organotropism suggested that local microenvironment of target organs (“soil”) provided an appropriate environment for tumor (“seed”) colonization and growth (7). The molecular mechanisms that govern the spread of cancers to specific organs such as the bone remains unclear, although technological advances have allowed examination of gene regulation in regards to organotropism. Such studies have found that tumor cells may acquire specific genetic phenotypes, with activation of specific cytokines and/or proteases which may govern metastasis to specific organs.

## Chemokines and Metastasis

Chemokines are a family of low molecular weight proteins which function in directing leukocyte cell chemotaxis to various tissues in both steady-state homeostasis and inflammatory conditions (8). There are approximately 50 chemokine ligands and 20 receptors identified to date. The chemokine family is divided into four subfamilies based on the arrangement of cysteine residues in the amino terminus of the chemokine ligand. The CCL family is comprised of chemokines whose first two cysteine residues are adjacent to each other and are designated CCL1–28. The CXCL group contains chemokines which have a single amino acid between the first two cysteine residues. There is one ligand, CX3CL1 (also called fractalkine) which has three amino acids between its first two cysteines, and there are two chemokines which are missing two of the four conserved cysteine residues referred to as XCL1 and XCL2 (9).

Chemokines are recognized by a family of receptors that belong to the superclass of seven transmembrane spanning G-protein coupled receptors (GPCRs). Nomenclature for the receptors is based on the subfamily of ligands they bind: CCR receptors activated by CCL ligands, designated CCR1–10; CXCR receptors bind to CXCL ligands, designated CXCR1–8; XCR is the sole receptor for XCL1 and XCL2 ligands; and the CX3CR1 receptor and CX3CL1 ligand form an exclusive pair. There is extensive promiscuity between the CC ligands and their receptors. There is some degree of promiscuity between the CXC ligands and their receptors, but it is not as extensive as that observed with CC ligands (8). A certain amount of redundancy is likely for chemokine receptors. This is an important point as pharmacological inhibitors of individual receptors may have limited efficacy due to the redundancy, and the tendency toward compensatory increases in expression of other chemokine family members.

The activation of chemokine receptors by their respective ligand(s) triggers a series of biochemical events characteristic of GPCR signaling in general with activation of heterotrimeric G proteins (Gαβγ). Most chemokine receptors have been shown to couple to multiple Gα proteins, each of which can engage and modulate the activity of effector proteins. These include Gi, that effects adenylyl cyclase results in the inhibition of the second messenger molecule cAMP; and Gq that effects phospholipase C ultimately resulting in the release of calcium and activation of calcium-dependent enzymes. Some chemokine receptors have been shown to activate G_12/13_ and thus stimulate exchange factor activity of the rho family of GTPases and subsequent F-actin reorganization. Like other GPCRs, chemokine receptors also signal through β-arrestin proteins. These effector pathways are thought to be involved in engaging the cell motility apparatus to allow cells bearing these receptors to respond to chemokine gradients.

G-protein coupled receptors have proven to be excellent pharmacological targets and amenable to high-throughput screening techniques (10). Chemokines receptors have no exception, with FDA approved drugs for CCR5 and CXCR4, and multiple drugs in the pipeline (10).

A landmark study in 2001 by Zlotnik and colleagues demonstrated that the chemokine system may explain the pattern observed for organotropic metastasis (11). In this paper, it was shown that breast carcinoma (BC) cells express CXCR4 and CCR7, and activation of these receptors resulted in reorganization, cell motility, and tissue-specific metastatic trafficking *in vivo*. Blockade of CXCR4 with neutralizing antibodies prevented the BC cell line MDA-MB-231 from metastasizing to the liver and lung as these organs express the CXCR4 ligand, CXCL12 (SDF-1). Since this paper was published, there have been many studies which examined the role of chemokines in the various stages of metastasis (9, 12–15).

## Bone Microenvironment

Bone is one of the major target sites for metastasis (16–19). Metastatic colonization of bone often causes pathological fractures, chronic pain, and neurological compression syndromes (20). The presence of metastatic lesions in bone is generally an indicator for poor prognosis (21, 22). Certain cancers, particularly BC, prostate carcinoma (PC), and multiple myeloma (MM) exhibit a tendency to metastasize to bone. In fact, 60–85% of patients with metastatic breast and PC harbor bone metastases (23). Bone marrow is one of the most frequent metastatic sites for late stage BC (24). Furthermore, the 5-year survival rate for breast cancer patients with metastasis to bone is under 10%. As with other bone-metastatic cancers, morbidity and mortality is generally associated with bone degradation due to osteolytic bone disease (OBD) (25). PC is the second leading cause of cancer-associated death in men. The 5-year survival rate of metastatic PC is only 23%. Bone is the major target organ of metastatic PC, accounting for about 90% of sites of distal metastasis for this cancer (26). The growth of PC in bone is predominantly osteoblastic as identified by radiographic analysis; however, the activation of osteoclasts (OCLs) is a necessary initial step in this process as well.

The bone microenvironment consists of mineralized extracellular matrix and several specialized cell types, including osteoblasts (OBs), OCLs, mesenchymal stem cells, bone marrow endothelial cells, hematopoietic cells, and adipocytes (27, 28). OBs are one of the major cellular components of bone and they are responsible for depositing collagen and the mineralized calcium phosphate (hydroxyapatite) that gives the bone extracellular matrix its structural strength. OCLs are large multinucleated cells which originate from the fusion of myeloid lineage precursors. They adhere to and break down bone by dissolving calcium phosphate crystals and proteolysis of the collagen matrix scaffold. The main growth factors involved in OCL maturation are colony stimulating factor-1 (CSF-1) and receptor activator of nuclear factor KB ligand (RANKL). The crosstalk between OBs and OCLs modulates the degree of bone synthesis and resorption. In addition to expressing RANKL to promote osteoclastogenesis, OBs can also secrete osteoprotegerin (OPG) which is a decoy receptor for RANKL. Generally, the ratio of RANKL/OPG dictates the level of OCL maturation and activity. Interestingly, OPG is associated with BC-mediated osteolysis and bone metastasis (29). In addition to its function as a negative regulator of RANKL signaling, OPG is able to engage other cell receptors, such as TNF-related apoptosis inducing ligand, Syndecan-1, and αVβ5 integrins and has been shown to modulate BC apoptosis and cell invasion (30, 31).

Indicators of OCL maturation include positive tartrate-resistant acid phosphatase (TRAP) staining and the presence of proteases, such as cathepsin K and matrix metalloproteinase 9.

Tumor cells within the bone generally promote osteolysis in the process of creating a favorable microenvironment as the bone matrix is rich in growth factors (fertilized soil as per the Paget analogy). Tumors after colonization of bone secrete pro-OCL maturation factors, such as parathyroid hormone-related protein, IL-11, and TNFα which stimulate OBs to increase RANKL and decrease OPG production (32). OCL maturation and bone resorption subsequently lead to bone pain and bone fragility. Activated OCLs then carry out bone resorption and in the process they release transforming growth factor β, insulin growth factors, and other growth factors that fuel the tumor cells to produce even more pro-osteolytic factors. This process results in a positive feedback condition often referred to as a “vicious cycle” that can lead to OBD (33). It has been increasingly recognized that in addition to factors such as CSF-1 and RANKL, chemokines are vital for OCL maturation and function. For a more comprehensive treatment of the molecular mechanisms governing OCL differentiation, maturation, and regulation of pro-tumorigenic growth factors, the reader is directed to more specific reviews on the subject (34, 35).

In this review, we focus on the role of chemokines in directly influencing components of the bone microenvironment which in turn enable osteolysis and tumor growth. The focus will be on BC, PC, and MM, cancer types for which bone is a prominent metastatic target. Particular attention will be paid to animal models where injection of tumor cells directly into bone allows for measuring the effect of chemokine blockade specifically on tumor growth as opposed to earlier steps in the metastatic cascade where chemokines are known to play a role.

## CCL2

CCL2 (also called monocyte chemoattractant protein/MCP-1) is the primary ligand for the CCR2 receptor which is normally expressed on monocyte/macrophages (8). This signaling axis has shown to be important for OCL formation under steady state conditions (36). BC progression is associated with an increase in CCL2 expression (37). One of the major consequences of CCL2 expression by BC cells is the recruitment of CCR2 positive myeloid cells to the primary tumor which facilitates metastasis in general. Increased CCL2 expression by BC cells, however, is also correlated with growth within bone microenvironment (38) Lu and Kang examined the role of CCL2 in bone metastasis using bone-tropic metastatic sublines of the human BC cell line MDA-MB-231 (38). Their work indicated ectopic overexpression of CCL2 increased bone-metastatic burden of a weakly bone-metastatic MDA-MB-231 subline by stimulating osteoclastogenesis. Conversely, blockade of CCL2 *via* a neutralizing antibody was shown to inhibit metastasis to bone of a strongly metastatic MDA-MB-231 subline (38). OB-derived CCL2 may also promote BC metastatic outgrowth in bone (39, 40). Several studies show OBs treated with conditioned media from BC cell lines increase in CCL2 which in turn can promote OCL maturation (as measured by TRAP positive staining and bone resorption) (39, 41, 42). Interestingly, OPG expression correlates with an increase in CCL2 in BC patients which may in part explain why it is associated with an increase in osteolysis and growth in bone (43).

The study of PC has been hampered by the lack of models which exhibit spontaneous metastasis to bone. However, there are a number of reports which highlight the role of chemokines in growth within bone. The importance of the CCL2–CCR2 axis in PC such as has been well documented and there is solid evidence for this pathway in mediating tumor growth in the bone microenvironent (44). PC patients who have advanced stage disease with bone metastasis have higher levels of plasma CCL2 levels than patients with early stage localized tumors (45). A study by Lu et al. showed that CCL2/CCR2 signaling has a dual role in PC progression in mediating both tumor invasion in bone and osteolysis (45). Consistent with BC, metastatic PC cells secrete CCL2 which accelerates OCL maturation and bone resorption *in vitro* and *in vivo*. Human PC3 cell conditioned media induces bone marrow cultures and RAW 264.7 cells to form OCLs *in vitro* and this effect is partially blocked by anti-CCL2 neutralizing antibodies (46). Depletion of CCL2 in PC3 cell rendered them unable to efficiently form tumors when implanted in SCID tibias (45). This function of PC expressed CCL2 in conditioning the bone microenvironment has been confirmed by several other reports (47–49). Preclinical studies have shown the effectiveness of CCL2 neutralizing antibodies in blocking PC tumor growth in bone both as a single agent and in combination therapy (46, 50–54). Recently, carlumab (CNTO-888), an CCL2 neutralizing antibody, was tested in Phase 2 clinical trials in patients with metastatic castration-resistant PC (NTC00992186) (55). Unfortunately, CCL2 levels were only transiently blocked and no stable inhibition of CCL2/CCR2 signaling was observed in these patients.

Lung carcinoma also tends to metastasize to bone, and there are several reports which implicate the chemokine system as being central to this process (56). As has been observed in other cancer models, lung tumor expression of CCL2 is associated with tumor growth in bone which likely mediated *via* an increase in OCL maturation. In one study, RNAi-mediated depletion of CCL2 in A549 carcinoma cells prevented osteoclastogenesis in tibias orthotopically injected with these cells and this had a modest effect of tumor cell proliferation within the bone (56). Oral squamous cell carcinoma (OSCC) and nasopharyngeal carcinoma and osteosarcoma are other cancers which are associated with bone pathology (57–59). These tumor types express high levels of CCL2 which have been shown to be responsible for OCL maturation and bone resorption by tumors generated by these cells (57, 59).

## CCL3

CCL3 (also called MIP-1α) is the principal chemokine ligand associated with MM growth in bone (60–62). MM is a malignancy of monoclonal plasma cells of post-germinal origin. They re-enter the bone marrow and disrupt the normal physiology of the bone microenvironment. As a result, common symptoms of MM include osteolysis and hypercalcemia. MM cells express high levels of CCL3 which was shown to promote OCL maturation in a RANKL-independent fashion *in vitro* (63). The *in vivo* role of CCL3 expression was examined in a xenograft model of MM (61). In this study, the human MM line ARH engineered to express antisense RNA against CCL3 was unable to efficiently promote OCL maturation or form tumors in bone. Similar results were observed when neutralizing antibodies against CCL3 were administered to mice bearing 5TGM1 MM tumors (64). The principal receptor for CCL3 is CCR1 which normally expressed on cells of the myeloid lineage (including OCLs) as well as NK cells and certain T-cell subsets (8, 65). CCR1 has been shown to interact with many other CCL family ligands, including CCL5 (RANTES), the mouse specific ligands, such as CCL6 and CCL9 (MIP-1γ), and human-specific ligands, such as CCL14, CCL15 (MIP-1δ), and CCL16. This ligand/receptor system shows a significant degree of promiscuity as CCL3 and CCL5 can activate CCR5 as well. Given the involvement of CCR1 and CCR5 in various diseases, such as rheumatoid arthritis and HIV/AIDS, there has been an intense effort to develop effective antagonists against these receptors (66, 67). The availability of CCR1- and CCR5-specific inhibitors enabled researchers to test relative involvement of the main CCL3 receptors in MM bone metastasis using the 5TMM experimental myeloma mouse model which is thought to recapitulate the human disease in several key aspects (68). Small molecule inhibitors BX471 and TAK779, which selectively inhibit CCR1 and CCR5, respectively, prevent OCL differentiation *in vitro*; however, only BX471 had a significant effect on 5TMM tumor burden and bone lesions *in vivo*. Since in this study, additional CCR1-specific inhibitors have confirmed CCR1 as being the critical CCL3 mediating OCL maturation (69, 70). A structurally unrelated CCR1 inhibitor CCX721 (an analog of the clinical compound CCX354) was able to show an even more potent effect in preventing MM and growth within the bone (71). These results are consistent with the model that CCL3 is a major pro-osteoclastogenic factor in MM and that these effects are mediated by CCR1.

## IL-8/CXCL8

Interleukin-8 (IL-8/CXCL8) is a member of the CXCL class of chemokines which bind to the receptors CXCR1 and 2 (8). IL-8 has pleiotropic effects on cancer cells and can impact many stages of tumor progression, including survival, proliferation, epithelial mesenchymal transition, invasion, and angiogenesis (72, 73). IL-8 also has potent pro-osteoclastogenic activity and has been identified as an osteolytic factor (74). BC overexpression of IL-8 has been observed in tumor samples and an elevated serum IL-8 level is associated with osteolysis and bone metastasis in BC patients (75, 76). A more recent study clearly demonstrates an important role for IL-8 in osteolysis associated with BC using the MDA-MB-231 model. Disruption of IL-8-mediated signaling through use of neutralizing antibodies slowed the growth of bone tumors in mice injected with MDA-MET BC cells (a bone-tropic subline of MDA-MB-231) (76). Strikingly, over 80% of mice harboring BC xenograft in bone that underwent treatment with anti IL-8 for 1 month showed no sign of tumor and these animals survived at higher rate (76). This study also showed that MDA-MB-231 parental cells engineered to overexpress IL-8 confer a greatly enhanced ability to stimulate osteoclast maturation and grow within the bone. BC cells also utilize OBs to generate more IL-8 within the bone microenvironment (40). MDA-MB-231 cells secrete Semaphorin 4D induces IL-8 (or the murine homolog of IL-8, CXCL5) expression in OBs within the bone marrow which in turn was shown to promote osteoclastogenesis and bone resorption (77). Interfering with Semaphorin 4D expression, prevented the upregulation of these ligands in OBs and dramatically reduced bone metastasis.

As is observed with BC, enhanced IL-8 expression correlates with bone metastasis in PC tumors (78). As mentioned in the previous section, human PC3 cell conditioned media can stimulate OCLs maturation and activity *in vitro* (46). This effect is partially blocked by individual treatment by blocking antibodies against CCL-2 or IL-8, however, a much stronger inhibition is observed in the presence of both antibodies indicating CCL2 and IL-8 activate parallel pathways and work synergistically to promote OCL maturation (46). Xenografts of bone-metastatic PC tumors were found to express high levels of IL-8 (49). One study showed that repression of IL-8 in PC cells overexpressing the N-myc downstream regulated gene 2 resulted in a decrease in PC bone metastasis and osteolysis (79). There is also evidence for IL-8 expression being important in osteolysis associated with lung carcinoma (80). A consistent pattern has emerged as carcinoma derived bone metastases use CCL2 and IL-8 in tandem within the bone microenvironment to promote osteolysis and growth.

## CXCL12/CXCR4

CXCL12 is secreted by several cell types within the bone marrow including OCLs and endothelial cells and this expression is well established in mediating osteotropism of several metastatic cancers (9). CXCR4 is highly conserved throughout evolution and is expressed on many cells. The role of CXCL12/CXCR4 signaling in tumor cell motility and invasion is well established (81–83). In addition to metastasis, CXCR4 signaling has been shown to mediate many pro-tumorigenic functions in cancer cells, including proliferation, survival, angiogenesis, and chemoresistance (84). As noted in the original Muller et al. paper, CXCR4 is associated with highly metastatic BC cells (11). The role of CXCR4 in metastasis has been the subject of extensive investigation since this initial report (15, 19, 25). CXCR4 is one of several genes that is consistently and universally upregulated in carcinoma cells which metastasize to the bone. Gene expression analysis of a subline of MDA-MB-231 selected *in vivo* for its ability to metastasize to bone revealed that CXCR4 is a major component of the bone-metastatic signature (85). CXCR4 has been validated in preclinical models as a target for BC metastasis (86–88). There are several studies which attempt to specifically address the role of CXCL12/CXCR4 in mediating tumor osteolysis and tumor growth within bone. The preponderance of evidence indicates that CXCL12/CXCR4 signaling is pro-osteolytic. CXCL12 has been recognized to stimulate migration of osteoclast precursors and upregulate several pro-osteoclastic genes (89, 90). There is at least one study, however, which shows the lack of CXCR4 results in an increase in OCL function (91).

Prostate carcinoma was one of the first cancer cell types shown to express CXCR4 (92–94). Tumor expression of CXCR4 is associated with poorer survival in PC patients (95). Several studies showed that interfering with the CXCL12/CXCR4 pathway directly influences PC growth within bone (96, 97). CXCR4-specific antagonists have been examined in preclinical studies including the small molecule pharmacological inhibitors Plerixafor (also known as AMD3100), CTCE-9908, and monoclonal neutralizing antibodies. Administration of these agents inhibits PC association with bone, decreases growth of bone-metastatic PC tumors and improves overall survival in mice (93, 96, 98, 99). However, there is another report in which inhibition of CXCR4 primarily blocks PC trafficking to bone without influencing tumor growth (99). This report noted that CXCR4 inhibition will, in addition to blocking tumor migration to bone, mobilize hematopoietic stem cell out of the bone marrow creating an even more favorable niche for tumor growth. It is likely that a balance of these different parameters will determine if blockade of CXCR4 will have a stronger effect on homing or growth within bone.

Inhibition of CXCR4 may have additional benefits in addition to anti-metastatic function as CXCR4 signaling can deliver anti-apoptotic signals to the cell and mediate resistance to standard chemotherapy such as doclitaxel (98). As described above, CXCL12 is a prominent bone marrow ligand and its involvement in recruitment of normal and cancerous trafficking to bone marrow has been studied extensively. Because inhibition of CXCR4 *in vivo* does not result in a complete inhibition of metastasis and disease progression, it suggests an additional CXCL12 receptor, CXCR7, which may function in a partially redundant fashion. Intriguingly, CXCR4 is able to regulate the expression of CXCR7 and overexpression of CXCR7 induces an increase IL8 in PC3 cells (100). These findings indicate a significant degree of crosstalk between different chemokine pathways.

Multiple myeloma cells express CXCR4 and as with BC and PC, there is evidence suggesting this receptor plays an important role in the expansion of MM in bone. Stimuli which enhance CXCR4 expression on MM (such as hypoxia) enhances colonization of bone (101). In addition to the receptor, CXCL12 is also highly expressed by MM cells (102). Neutralization of CXCL12 prevents both homing and growth of MM within bone. Direct intratibial injection of the MM line RPMI-8226 resulted in a modest level of bone loss which correlated with CXCL12/CXCR4 activity (103, 104).

Lung carcinoma metastasis of bone also involves CXCR4/CXCL12 signaling, however, the relative importance of pathway in survival, homing, and osteolysis is not clear (105, 106). Recently, CXCR4 was discovered to be a biomarker for NSCLC bone metastasis and presumably expression of this receptor facilitates homing to the CXCL12 expressing bone marrow as observed in other metastatic cancers (107, 108). As is observed with other cancers, CXCR4 expression correlates with “cancer stem-cell” properties which include a propensity for tumor initiation and metastasis (109, 110). However, one study using AMD3100 showed that NSCLC metastatic colonization of bone did not require CXCR4 activity, however, outgrowth of metastases and subsequent osteolysis was dependent on the receptor (106).

## Other Chemokines

### CCL4

Other cell types within the bone marrow stroma can contribute to tumor growth. The murine cell line 4T1 model is used to study BC in the context of an immunocompetent animal. A subline (named 4T1.3) was selected for bone metastasis by repeated cycles of isolation of mammary fat pad injection and isolation from bone marrow (111). DNA microarray analysis of this subline revealed higher levels of CCL3 and CCL4 (both ligands for CCR5) expression relative to parental line (111). It was found that BC expression of CCL4 activates CCR5 on bone marrow fibroblasts which results in their expression of connective tissue growth factor/CCN2 (111). This, in turn promotes BC cell proliferation and subsequent growth of tumor within the bone. Knockdown of CCL4 had no effect on cancer cells *in vitro* but limited in its ability to form tumors in bone marrow *in vivo*, clearly highlighting the role of the microenvironment for promoting tumor expansion. A similar phenotype to knockdown of CCL4 was observed in CCR5 loss of function experiments. When 4T1.3 cells were injected into the bone of either CCR5 knockout mice or mice treated with a CCR5 antagonist, they failed to grow efficiently. The ongoing efforts to develop CCR5-specific antagonists are, therefore, expected to have efficacy against metastatic tumors that utilize CCL3 and CCL4 to condition the bone microenvironment.

### CCL15

Renal cell carcinoma (RCC) is a relatively rare tumor of the kidney which often metastasizes to bone. Kominsky et al. showed that metastatic RCC expressed higher levels of CCL15 than patient matched primary tumors (112). The study proposed that CCL15 conditions the bone microenvironment by directly stimulating OCL differentiation. As CCL15 is a ligand for CCR1, this finding is consistent with MM model, whereby CCL3 (the principal CCR1 agonist) is necessary for OCL maturation (63). Recombinant CCL15 was confirmed to be a pro-osteoclastogenic factor (113). In addition to CCR1 and CCR5, mature OCLs express significant levels of an additional MIP-family ligand receptor, CCR3 (112). The relative roles of CCR3 (as opposed to CCR1) in metastasis and tumor growth in bone were not addressed and its potential importance in bone metastasis *in vivo* remains to be studied.

### CXCL1/2

It was discovered that PC tumors utilize other ligands for CXCR1/2 in addition to IL-8 during the process of bone metastasis (114). Bone marrow adipocytes were shown to be a source of CXCL1 and CXCL2, and these chemokines were shown to drive OCL maturation. Recombinant CXCL1 can modestly increase TRAP positive cells and adipocyte conditioned media stimulated osteoclast formation *in vitro* in a CXCL1 and CXCL2-dependent fashion. Consistent with this role for adipocytes, a high fat diet was observed to enhance PC-mediated bone osteolysis (114).

### CX3CL1

Fractalkine (CX3CL1) is also associated with bone metastasis. CX3CL1 is predominantly expressed in tissue macrophages and certain lymphoid subsets and it exists as a membrane bound precursor which is ultimately processed into the mature soluble ligand. Blockade of proteolytic CX3CL1 cleavage and release has been suggested as an alternative therapeutic avenue as a means of disrupting the chemokine gradient that attracts CX3CR1-bearing tumor cells to the bone (115). It was demonstrated that CX3CR1 signaling mediates metastasis of BC specifically to the bone marrow (116). In this study, BC cells that expressed CX3CR1 showed a high preference for metastasizing to the bone. CX3CL1 is expressed on endothelial cells and stromal cells within the bone marrow. MDA-MB-231 metastasis to bone was dramatically reduced in CX3CL1 null mice, whereas metastasis to adrenal glands was relatively unaffected. CX3CR1 is important for mediating adhesion and extravasation to bone marrow epithelium. Recently, a novel small molecule inhibitor of CX3CR1 was effective in preventing skeletal metastasis in a BC pre-clinical model (117). CX3CR1 is also expressed by OCL precursors and treatment with CX3CL1 accelerates their differentiation contributing to bone resorption which suggests that increased production of this chemokine may play a role in osteolysis as well as trafficking (118).

## Therapeutic Targeting of Chemokines in Bone Metastasis

Current therapies for treating bone metastasis attenuate osteoclast activity. These include bisphosphonates which are taken up by OCLs and interfere with their ability to lyse bone and anti-RANKL antibodies (such as denosumab) which prevent OCL maturation. Interestingly, certain bisphosphonates (such as zoledronic acid) may impart some of their benefit by acting on chemokine signaling (97, 119). Discovery of novel therapies which target bone-metastatic PC, such as *radium*-223 dichloride (Xofigo^®^) a calcium-mimetic agent that specifically targets bone lesions, has been shown to have an impact on survival rates for this disease (120). The current treatments, however, are mainly palliative and are not very effective at slowing tumor growth within bone. Chemokines are an attractive target for metastatic bone cancer and OBD. Not only are chemokines involved in most steps of the metastatic cascade, including survival, angiogenesis, invasion, and trafficking to bone but are also strongly associated with OBD and growth. Chemokine receptors are also amenable to inhibition by small pharmacological compounds. The relevant chemokines and the roles they play during bone metastasis are summarized in Table 1.

**Table 1:** Summary of chemokine ligands and receptors used by tumor cells within the bone microenvironment.

Cancer type	Chemokine ligand/receptor (reference)	Function
Breast carcinoma (BC)	CCL2 (38)	Osteolysis
	CCL4 (111)	Communication with bone fibroblasts/growth within bone
	IL8/CXCL8 (75)	Osteolysis and tumor growth in bone
	CXCR3 (121)	Osteolysis and growth in bone

Prostate carcinoma	CCL2 (45, 46)	Bone resorption, tumor growth in bone
	CCR5 (122)	Osteolysis
	CXCL1 (114, 123)	Paracrine action on endothelial cells and osteoblasts, osteolysis
	CXCL2 (114)	Osteolysis
	CXCL8 (46, 49)	Osteolysis, tumor growth in bone
	CXCR4 (92, 93)	Trafficking and migration to bone, tumor growth in bone

Multiple myeloma	CCL3 (60)	Osteolysis
	CCR1 (68, 69)	Osteolysis, tumor growth in bone

Although many different chemokine systems are involved in these processes depending on the tumor type studied, a broad consensus has emerged. There is clear evidence that carcinomas, such as BC, PC, lung, and OSCC often utilize both CCL2 and IL-8 to condition the bone microenvironment and promote OBD (49, 75, 76). These tumors overexpress CCL2 and IL-8 and stimulate OB production of these chemokines to act on OCLs in a paracrine fashion. These chemokines synergize in OCL maturation. *In vivo* blockade of both of these pathways may prove to be very effective on treating bone-metastatic cancers.

The role of CCL3 and its receptor CCR1 in bone metastasis have been best characterized in the context of MM and blockade of this receptor has promising effects in pre-clinical animal cancer models. Lending weight to this therapeutic avenue is the fact that CCL15, another CCR1 agonist, is associated with osteoclastogenesis and bone metastasis (112). This pathway, however, is complex and although most studies indicate CCR1 as being the major receptor expressed on OCLs, there might be some degree of redundancy as CCR1, CCR3, and CCR5 share many of the same pro-osteoclastic ligands. It will be interesting to determine if pharmacological inhibition of one receptor results in the upregulation another as a potential mechanism of chemoresistance.

The role of CXCL12/CXCR4 in cancer cell trafficking to bone has been explored extensively; however, there is a potential function for this pathway in modulating OCLs and bone resorption. Blockade of CXCR4 in most tumor models prevents OCL maturation and osteolysis. However, it is has been reported that mice reconstituted with CXCR4 null hematopoetic cells display more OCLs and osteolysis (91) It was noted in this paper that CXCR4 null macrophages proliferated and differentiated into OCLs at a higher rate. The tumor model used in this study was the B16 melanoma cell line which perhaps also suggests that the role of CXCR4 in osteolysis is dependent on the context of other factors secreted by tumor cells. Regardless, the conflicting data give pause to potential use of CXCR4 antagonists in treating metastatic bone cancers.

**Figure 1:**
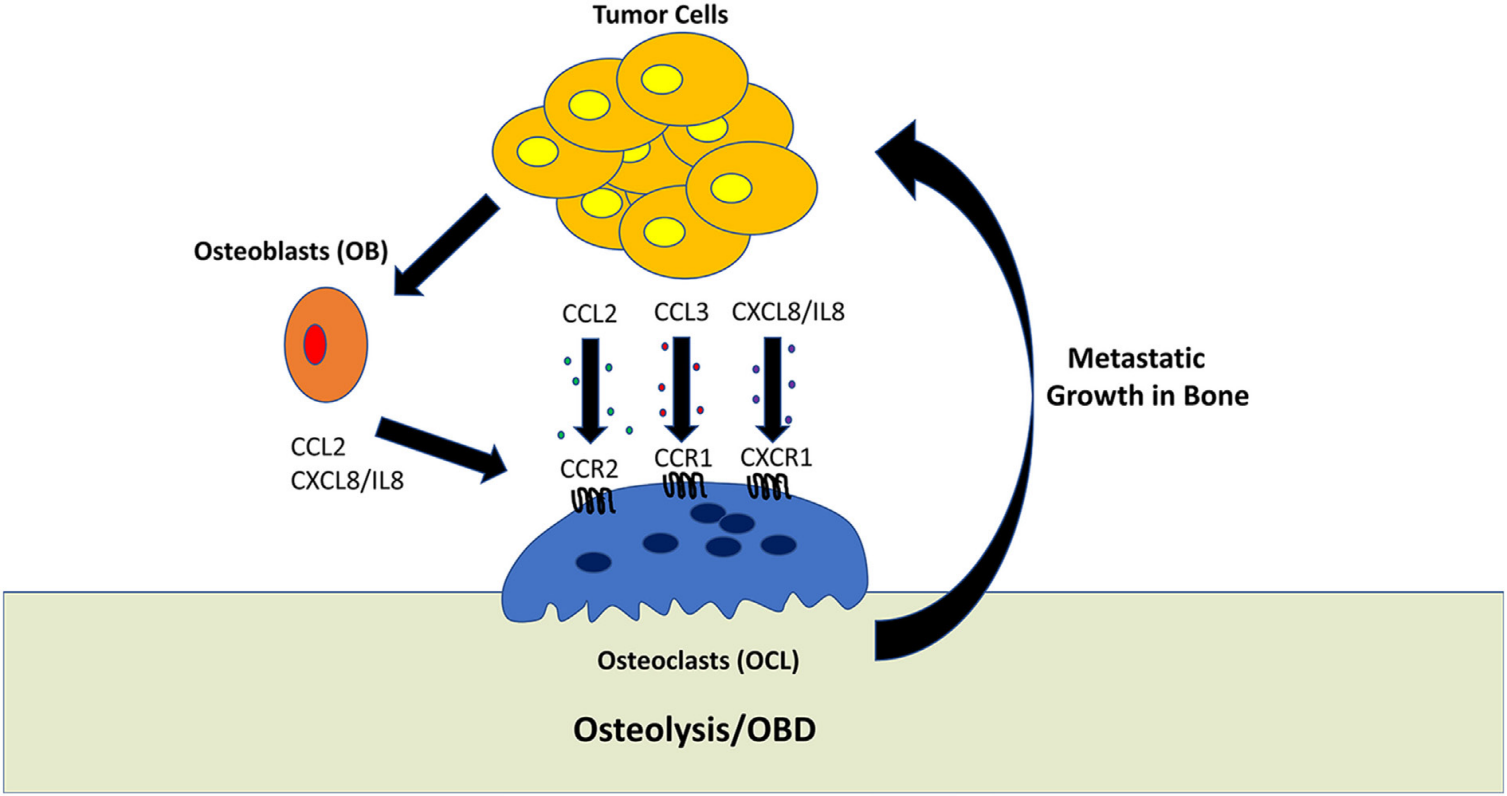
Role of tumor-derived chemokines in osteolysis. Osteoclasts (OCL) are multinucleated cells which mediate the resorption of bone. This process generally favors tumor growth within the bone microenvironment. Tumor cells can directly promote OCL activity by secreting the chemokines, such as CCL2, CCL3, and CXCL8/IL-8. In addition, tumor cells can stimulate osteoblasts to activate OCL differentiation and activation.

This review would not be complete without mentioning that the study of bone metastasis (and metastasis in general) is hampered by the death of human cell lines which are capable of exhibiting consistent and reliable metastatic behavior in mouse models. Even the cell lines that do provide metastasis in mouse models often fail to display the full spectrum of malignant features. Most pre-clinical studies have been carried out using only a limited number of cell lines or genetic mouse models of cancer. Novel *in vitro* and *ex vivo* 3D culture systems are currently being developed to augment the study of tumor cells within the bone microenvironment (124, 125). However, even with these limitations, many of the findings discussed have been validated by the fact that the same chemokine systems appear to be involved in mediating bone metastasis regardless of the cancer of origin, in particular CCL2/CCR2, CCL3/CCR1, IL-8/CXCR1, and CXCL12/CXCR4. A summary of these findings are shown in Figure 1.

The use of neutralizing antibodies against soluble ligands and small molecule pharmacological inhibitors that target the relevant chemokine receptors have yielded encouraging results in a number of pre-clinical models (Table 2). For those patients diagnosed after metastasis has occurred the efficacy of tumor anti-migration/invasion strategies which may depend on the frequency of secondary metastatic events. Targeting the chemokine system to slow or prevent OBD has the potential for palliative, if not curative results. Stopping the vicious cycle induced by cancers in the bone may not only slow the formation of osteolytic lesions but may also cause the tumor cells to slow their growth.

**Table 2:** Summary of chemokine antagonists used in preclinical animal models that show efficacy in treating bone metastasis.

Chemokine receptor/pathway	Cancer model (reference)	Chemokine antagonist used in study
CCR1	Myeloma (68)	BX471
	Myeloma (71)	CCX354 (CCX721)

CCL2/CCR2	Breast (38)	CNTO-888 (anti-CCL2 antibody)
	Prostate (47, 51–54)	CNTO-888/C1142 (anti-CCL2 antibody)

CCR5	Myeloma (68)	TAK-779
	Prostate (122)	Maraviroc

CXCL8/CXCR1	Breast (76)	Anti-CXCL8/IL-8 antibody

CXCL1-5/CXCR2	Breast (126)	Anti-CXCL5 and Anti CXCR2 antibodies
	Prostate (127)	Anti-CXCL1 antibody

CXCL10/CXCR3	Breast (121)	Anti-CXCL10 antibody
	Melanoma (121)	Anti-CXCL10 antibody

CXCL12/CXCR4	Myeloma (102)	Plerixafor/AMD3100
	Breast (86)	CTCE-9908
	Breast (88)	POL5551
	Prostate (93)	Anti-CXCL4 antibody
	Prostate (96)	Plerixafor/AMD3100 and CTCE-9908
	Prostate (98, 99, 128)	Plerixafor/AMD3100
	NSCLC (106)	Plerixafor/AMD3100

CXCL16/CXCR6	Prostate (127)	Anti-CXCL16 antibody

CXCR7	Myeloma (129)	POL6926

CX3CL1/CX3CR1	Breast (117)	JMS-17-2

*These anti-chemokine agents demonstrated an inhibition of either metastasis to bone, osteolysis, or tumor growth within bone*.

Given that multiple chemokine ligand/receptor pairs may be driving bone metastasis, a combinatorial approach targeting multiple chemokine pathways simultaneously, may be required for effectively preventing bone metastasis. In most of the *in vivo* pre-clinical model studies, targeting a single receptor fails to completely inhibit bone metastasis and the effect on prolonging animal survival is modest. It is possible in these cases that other chemokine receptors are involved in the residual migration, invasion, and growth within the bone. For example, the expression of several chemokines by BC cells (CCL2, CCL4, IL-8) have been shown in various models to promote growth within bone. Blockade of one of these results in upregulation and/or usage of one of the others to carry out the pro-metastatic function. Advanced cancers are notorious for being able to develop chemoresistance and switching from one chemokine system to another would seem to be a likely strategy adopted by malignant tumors. To target multiple chemokine ligand/receptor pairs would require combinations of anti-chemokine agents or use of pharmacological inhibitors which have a broader target range. Either strategy may be advantageous in this scenario. Given the diversity of chemokines used by a given tumor type to metastasize to bone, it may be necessary to profile individual patients for chemokine ligand/receptor expression to determine which agent is likely to be most effective for a given patient. The chemokine expression profile would also have to be monitored over treatment time as it is the possible tumor cells which may acquire resistance by switching ligand expression.

## Author Contributions

The author confirms being the sole contributor of this work and approved it for publication.

## Conflict of Interest Statement

The author declares that the research was conducted in the absence of any commercial or financial relationships that could be construed as a potential conflict of interest.

## Abbreviations

BC, breast carcinoma; CTCs, circulating tumor cells; MM, multiple myeloma; OBD, osteolytic bone disease; OB, osteoblasts; OCL, osteoclasts; PC, prostate carcinoma; IL-8, interleukin-8.
